# The Use of Dynamic Neck Lymphaticovenous Anastomosis in the Adjunctive Treatment of Alzheimer's Disease: A Retrospective Case Series

**DOI:** 10.1055/a-2863-3797

**Published:** 2026-07-07

**Authors:** Xuhui Chen, Johnson Boey, Kou Wei, Chen Ding, Paloma Malagón, J. P. Hong, Zhegang Zhou

**Affiliations:** 1 74573 Department of Neurology, Peking University Shenzhen Hospital, Shenzhen, Guangdong, China; 2 569261 Department of Podiatry, SingHealth Duke-NUS Academic Medical Centre, Singapore, Singapore, Singapore; 3 85075 Department of Podiatric Medicine and Surgery, The University of Western Australia Faculty of Medicine Dentistry and Health Sciences, Perth, Australia; 4 66310 Department of Hand Foot and Microsurgery Reconstruction Surgery, The First Affiliated Hospital of Shandong First Medical University, Jinan, Shandong, China; 5 Department of Plastic Surgery, Hospital Germans Trias I Pujol, Universitat Aut noma de Barcelona, Badalona, Barcelona, Spain; 6 35029 Asan Medical Center, Department of Plastic Surgery, University of Ulsan, Seoul, Seoul, Korea; 7 74573 Hand and Microsurgery Department, Peking University Shenzhen Hospital, Shenzhen, China

**Keywords:** Research, lymphedema, head and neck, others, neurodegenerative, Alzheimer's disease, dementia, lymphaticovenous anastomosis, cervical lymphatic vessels

## Abstract

**Background:**

Lymphovenous anastomosis (LVA) has recently emerged as a potential method for the symptomatic management of Alzheimer's disease (AD). Building on this, we conceptualize the “dynamic neck lymphovenous anastomosis” (DN-LVA) to illustrate the versatility of our technique. DN-LVA utilizes a vein graft that traverses through the sternocleidomastoid muscle to facilitate the drainage of lymphatic fluid from the deep cervical lymphatic vessels to the external jugular vein.

**Methods:**

This study presents a retrospective analysis of patients who underwent DN-LVA between November 2024 and January 2025. The clinical outcome was evaluated through positron emission tomography/computed tomography (PET/CT) imaging and cognitive assessment tools, which included Mini-Mental State Examination (MMSE), Montreal Cognitive Assessment (MoCA), Neuropsychiatric Inventory (NPI), and Clinical Dementia Rating (CDR). The vein graft was harvested from the lower limb.

**Results:**

Five subjects were recruited into this study. Postoperative PET/CT scans revealed up to a 6.9% increase in glucose metabolism in patient 3, a decrease of 12.3% in amyloid deposits, and a 4.7% reduction in tau deposits in patient 5 and patient 4, respectively. Clinically, MMSE and MoCA scores were improved by 1.8 and 1.4, respectively. NPI scores decreased by 5.8 across five overall, and the CDR remained unchanged in all subjects. All grafts demonstrated vessel patency, and no postoperative complications were observed.

**Conclusion:**

DN-LVA appears to be a safe, minimally invasive procedure with potential cognitive and metabolic benefits in AD. Further studies with larger cohorts and longer follow-up are warranted to confirm these preliminary findings.

## Introduction

Alzheimer's disease (AD) is a neurodegenerative disorder characterized by progressive memory loss, cognitive decline, and alterations in behavior. 1 2 As the condition progresses, patients frequently exhibit confusion, language deficits, and changes in mood or personality, ultimately resulting in the loss of the capacity to perform daily activities. Although amnesia and associated symptoms generally develop progressively over a span of years, early diagnosis and appropriate intervention can alleviate symptoms and maintain quality of life.

The pathogenesis of AD is primarily linked to the accumulation of amyloid-beta (Aβ) peptides and neurofibrillary tangles (NFTs) formed by hyperphosphorylated tau proteins. 3 The diagnosis of AD is based on the assessment of biomarkers, neuroimaging, and cerebrospinal fluid (CSF), alongside cognitive and neurological evaluations. 4 5 Despite advancements in understanding, no curative therapy is available, and existing treatments primarily focus on pharmacologic interventions to alleviate symptoms rather than to stop disease progression. 5 Recent advances in understanding brain fluid clearance have underscored the significance of the glymphatic system and meningeal lymphatic vessels (LVs) as essential pathways for the removal of macromolecules and neurotoxic proteins from the brain parenchyma, indicating a possible involvement in the mechanisms of neurodegenerative diseases, such as AD. 6 7 8 9

These insights have prompted the exploration of the lymphovenous anastomosis (LVA) technique to enhance lymphatic drainage in AD. 10 11 12 13 Multiple techniques, including lymph node-to-vein anastomosis, lymphatic vessel-to-vein anastomosis, octopus anastomosis, lymphatic adipose tissue-to-vein anastomosis, and thoracic duct decompression, have demonstrated promising preliminary results; however, additional validation is necessary. 14 15 16 17 Against this backdrop, this paper evaluates the feasibility, safety, and clinical outcomes of our dynamic neck lymphovenous anastomosis (DN-LVA) technique, a supermicrosurgical approach aimed at improving CSF and interstitial fluid clearance for symptomatic management of AD.

## Methods

### Patient Selection

This retrospective case series analyzed patients who had previously received medical management for AD and subsequently underwent DN-LVA from November 2024 to January 2025. The inclusion criteria comprised (1) individuals under 80 years of age; (2) possession of at least an elementary school education; (3) diagnosis of AD, in accordance with the National Institute of Aging–Alzheimer Association (NIA-AA) criteria, 5 with no significant improvement after 6 months of medical management; (4) continuous memory decline for a minimum of 1 year; and (5) availability of stable and reliable caregivers to ensure accurate pre- and postoperative data collection. This study excluded dementia of other origins, including vascular dementia, Lewy body dementia, and Parkinson's disease, as well as sensory impairments that hinder neurological evaluation, psychiatric disorders such as depression or psychosis, substance abuse, and non-compliance with postoperative treatment protocols.

Informed consent was obtained from all participants, with at least one family member or caregiver providing consent following a detailed explanation of the procedure's aims, benefits, and risks. The study obtained approval from the Institutional Review Board and the Ethics Committee at Peking University Shenzhen Hospital.

### Clinical Evaluation

Participants who fulfilled the inclusion criteria during recruitment were evaluated for their suitability for LVA. The initial evaluation includes cognitive assessment, positron emission tomography/computed tomography (PET/CT) scans, and CSF analysis. The evaluation of cognitive status was conducted using the Mini-Mental State Examination (MMSE), Montreal Cognitive Assessment (MoCA), Clinical Dementia Rating (CDR), and Neuropsychiatric Inventory (NPI) at baseline and 1 month postevaluation to assess the efficacy of our modified technique.

The preoperative PET/CT (Discovery MI DR, Shanghai, China), performed by an independent radiologist, was employed to evaluate glucose metabolism, as well as amyloid and tau deposition, using three 18-Fluorine [ ^18^ F]-based radiolabeled tracers: Fluorodeoxyglucose (FDG), florbetapir (AV45), and flortaucipir (AV1451). To assess glucose metabolism, a fasting period of at least 4 hours is necessary before administering 185 MBq (5 mCi) of [ ^18^ F]-FDG. In the study involving [ ^18^ F]AV-45 and [ ^18^ F]AV-1451, 370 MBq (10 mCi) of radiotracers were administered intravenously without fasting requirements. Standardized uptake value ratios (SUVr) were computed with the cerebellar cortex serving as the reference point. Regional average SUVr was calculated using 20-minute time intervals following various configuration start times after the administration of different radiotracers. Image data acquisition was performed at specific time intervals: 30 to 60 minutes after the injection of [ ^18^ F]-FDG, 50 to 70 minutes after [ ^18^ F]AV-45, and 80 to 100 minutes after [ ^18^ F]AV-1451. These regions encompassed the frontal, parietal, temporal, occipital, and insular cortices; the association cortex; the hippocampus; the basal ganglia; and the cingulate gyrus. Increased SUVr values of ^18^ F-FDG signify enhanced glucose metabolism, whereas higher SUVr values of ^18^ F-AV45 and ^18^ F-AV1451 denote the accumulation of Aβ and tau, respectively. The PET/CT imaging was repeated 1-month postoperatively to evaluate the efficacy of our modified technique.

CSF samples were collected via lumbar puncture between the third and fourth lumbar intervertebral spaces while the patient was placed in the lateral decubitus position following local anesthesia (2% lidocaine). A 5-mL sample was centrifuged at 1,600 g for 12 minutes at 4 °C and stored at −80°C. Biomarkers Aβ40, Aβ42, neurofilament light, phosphorylated tau (p-Tau) 181, and p-Tau 217 were measured using Simoa technology (Simoa NEUROLOGY 4-PLEX E and Simoa HD-X Analyzer, Quanterix Corp.).

### Surgical Protocol


**Video 1 Supplementary video.**


**Fig. 1:**
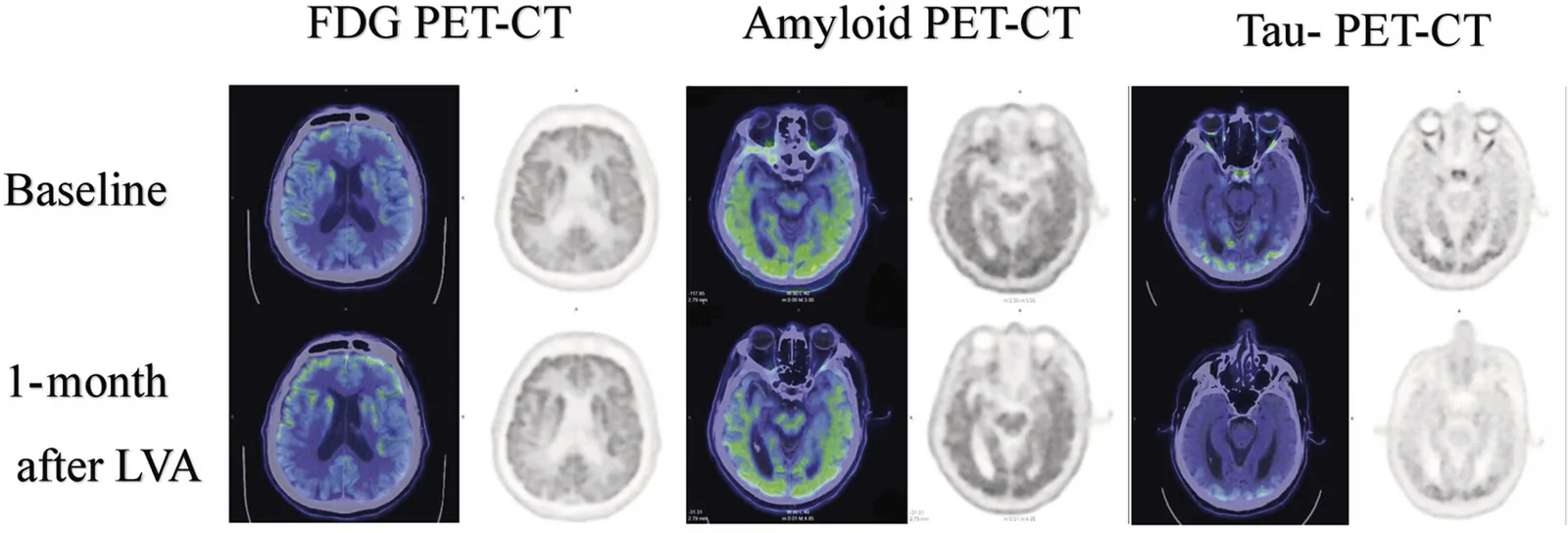
About 0.2 mL of indocyanine green (ICG) is administered via injection into the vestibule and nasal conchae of the nasal cavity.

A comprehensive preoperative assessment was conducted to determine the suitable LVs and veins for the venous graft harvest intended for LVA. High-frequency ultrasound was employed to identify the sternocleidomastoid muscle (SCMM) and to map the lymphatic and venous structures in the cervical region, encompassing the internal jugular vein (IJV) and external jugular vein (EJV). Fluorescence-assisted imaging was conducted 30 minutes following the administration of 0.2 mL of indocyanine green (ICG; Dandong YiChuang Pharmaceutical Co. Ltd., China) via injection into the vestibule and nasal conchae of the nasal cavity ( Fig. 1) . The injection site was modified from the cervical region, as shown in Supplementary video (available in the online version only), to the nasal cavity to minimize contamination of the surgical field while providing improved-quality ICG lymphographic images. ICG lymphography enabled localization of dilated LVs measuring approximately 0.2 to 0.8 mm in diameter. In addition, a vein finder was used to identify suitable superficial veins within the dorsal venous arch for the harvest of autologous vein graft

**Fig. 2:**
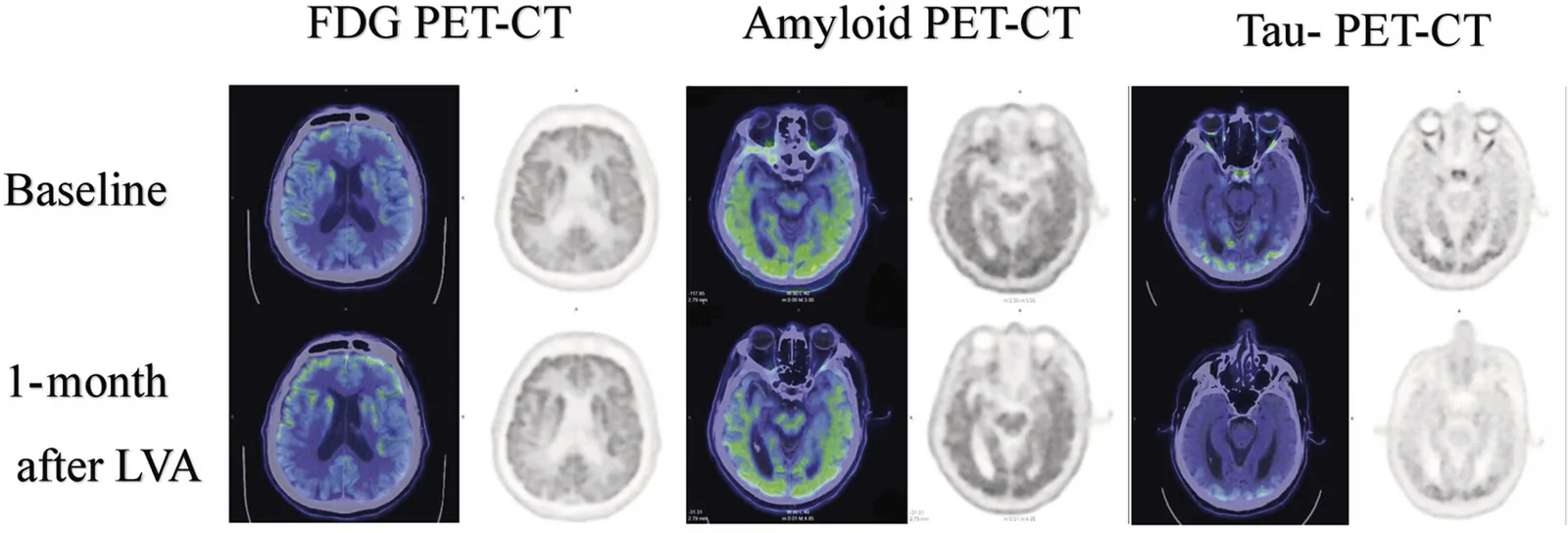
A transverse incision is performed over the SCMM, and the location of EJV is identified by ultrasonography. EJV, external jugular vein; SCMM, sternocleidomastoid muscle.

**Fig. 3:**
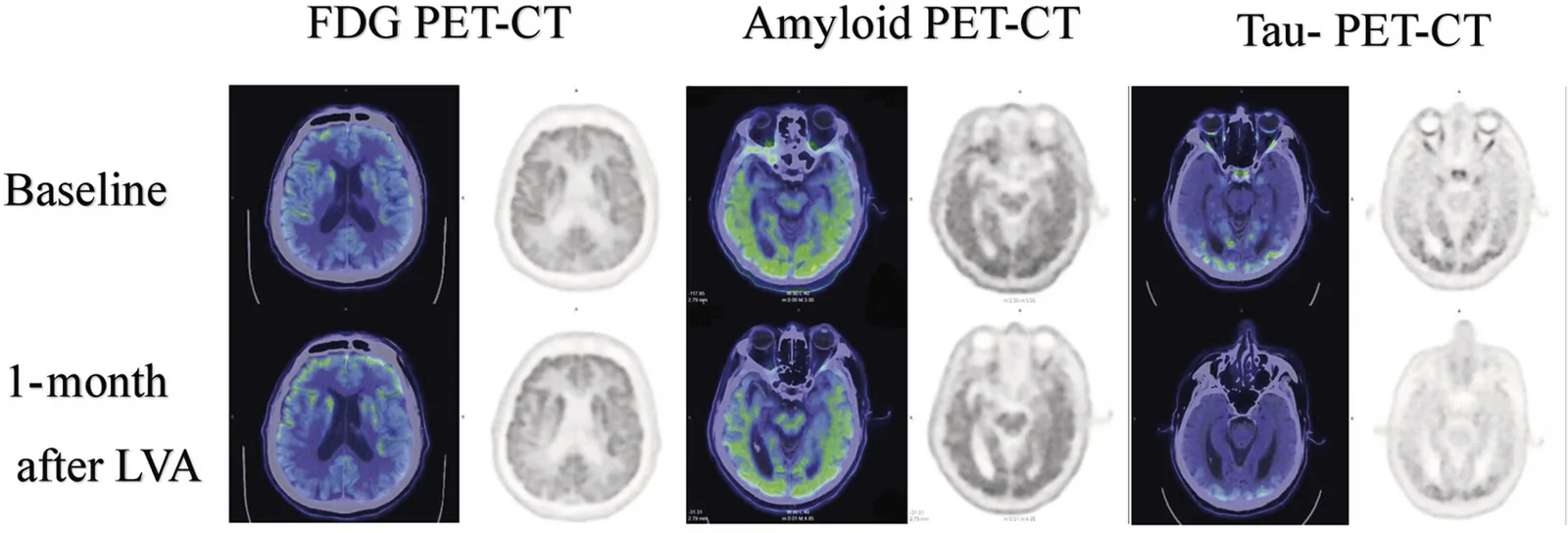
A vein finder is used to visualize the superficial vein network of the forefoot, including the dorsal venous arch and the corresponding dorsal digital veins. A 2.5-cm superficial vein with three tributary branches was harvested from the dorsal aspect of the foot.

**Fig. 4:**
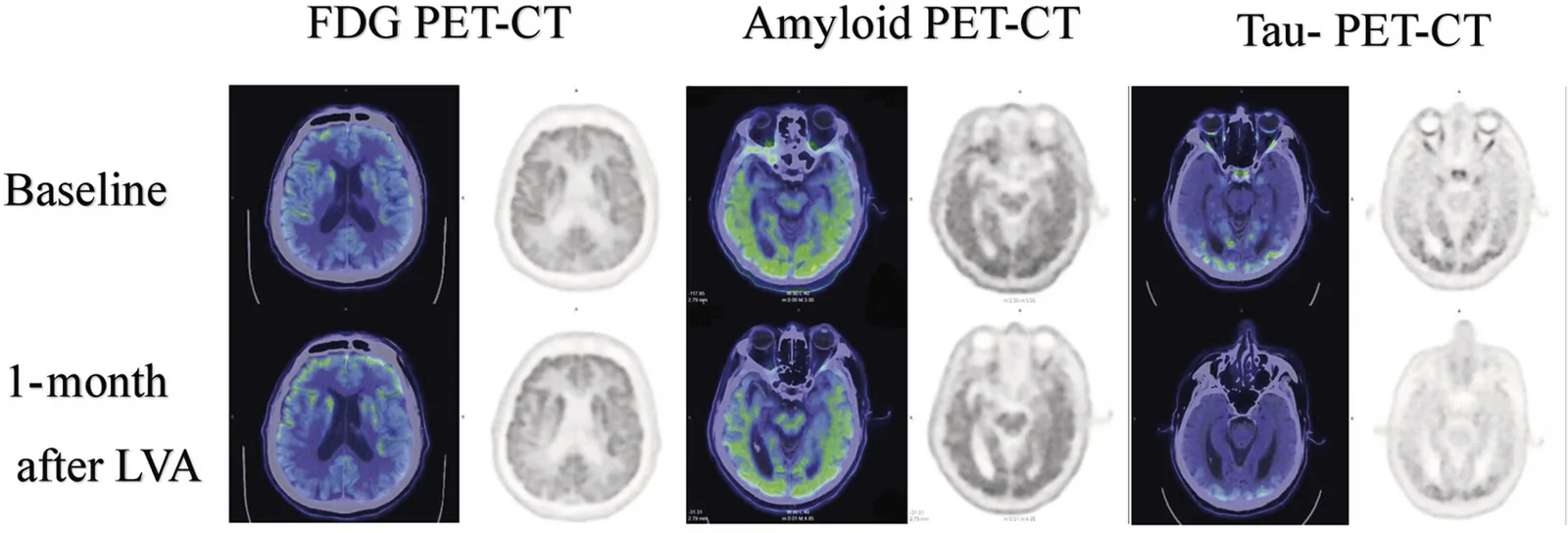
The venous graft harvest from the dorsalis pedis vein (indicated by asterisk, *) is positioned in the center of the surgical field. An end-to-side anastomosis of the proximal segment of the vein graft to the EJV, while three end-to-end anastomoses of the distal segment of the vein graft to three corresponding LVs. EJV, external jugular vein; IJV, internal jugular vein; LV, lymphatic vessel; SCMM, sternocleidomastoid muscle.

**Fig. 5:**
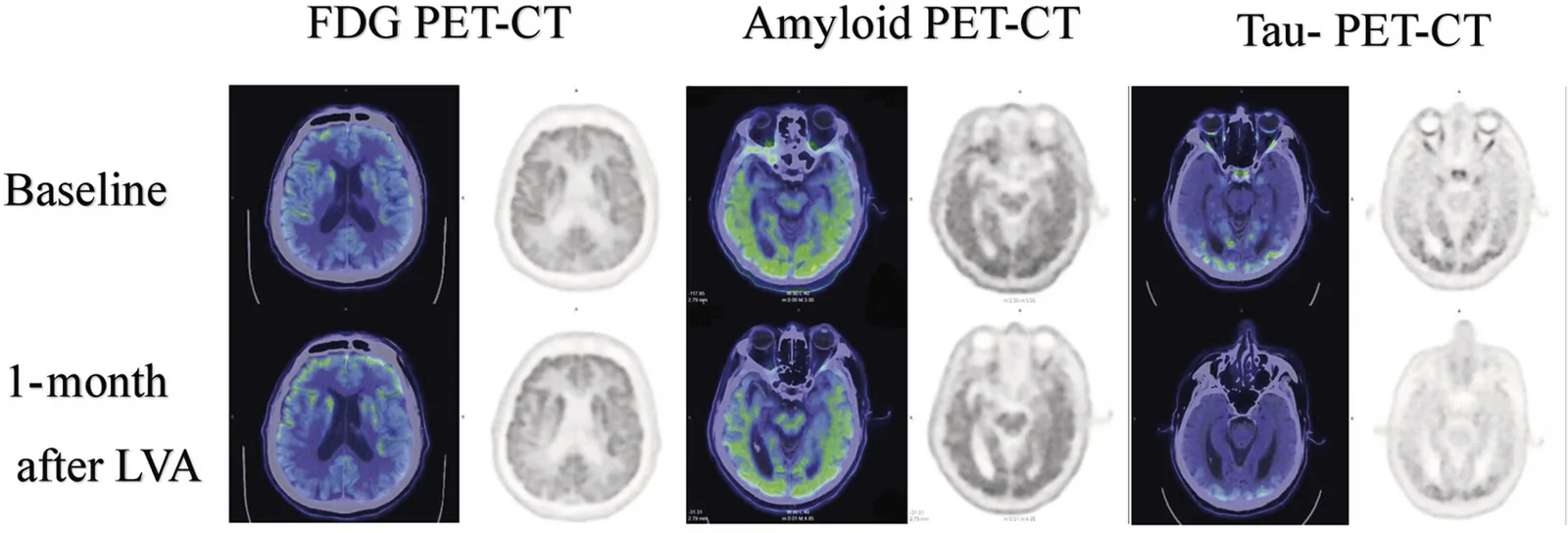
ICG lymphography is performed to confirm vessel patency between the LVs at the distal end of the vein graft and the EJV at the proximal end. EJV, external jugular vein; ICG, indocyanine green; LV, lymphatic vessel.

A transverse incision of 3 to 4 cm was performed over the sternocleidomastoid muscle, extending towards the anterior margin of the EJV ( Fig. 2 ). The platysma was dissected to expose the EJV and its branches, while the sternocleidomastoid muscle (SCMM) was dissected longitudinally to create a slit and separated until the IJV was identified. The IJV was retracted to expose the underlying LVs. We used fluorescence-assisted microscopy with the Zeiss Kinevo 900 (Carl Zeiss Meditec AG, Germany) to identify LV and measure the distance between LVs and the EJV. Vein finder was used to locate superficial vein measuring between 2.5 to 3.5 cm in length and 1 mm in thickness, along with three tributary branches, in the lower limb ( Fig. 3 ). The donor vein graft was placed through the opening in the SCMM at the distal aspect to connect to the superficial EJV ( Fig. 4 ). An end-to-side anastomosis was performed between the vein graft and the EJV proximally, while an end-to-end anastomosis was carried out with the deep cervical LVs distally ( Fig. 4 ). Patency was confirmed intraoperatively by direct visualization of lymphatic fluid entering the vein graft and through ICG lymphography ( Fig. 5 ). The procedure was performed on the opposite side. Patients were discharged within 72 hours, and sutures were removed 1 week postoperatively.

## Results

The study included five patients, comprising two males and three females. The average age was 62.4 ± 4.7 years. Additional demographic and clinical data are provided in Table 1 . All patients demonstrated memory loss, impaired decision-making abilities, and challenges in communication. The NIA-AA stages varied from 4C to 6D. A total of 76 LVAs were performed on five patients, with an average of 8 to 20 anastomoses per patient. Venous grafts ranging from 2.5 to 3.5 cm, as measured between the EJV and deep cervical LVs, were harvested from the lower limb and used in all patients. The diameter of the deep cervical LVs ranged from 0.2 to 0.8 mm. All anastomoses demonstrated patency, as confirmed by direct visualization or ICG lymphography. Venous reflux into the lymph vessels was not observed in any of the cases. The average surgical duration was 6.5 hours, with a range of 6 to 8 hours. No postoperative delirium or surgical complications were reported. No perioperative complications were reported in the neck or lower limb regions. All patients were released within 72 hours. The follow-up ultrasonography examination of the donor graft indicated the absence of embolism.

**Table 1:** Demographics of patients treated with dynamic neck lymphovenous anastomosis

	Subject 1	Subject 2	Subject 3	Subject 4	Subject 5
Age (years)	55	61	64	65	67
Gender	Female	Female	Male	Female	Male
Education level	Primary	Junior high	Junior high	High school	University
Comorbidities	Hyperlipidemia, sleep apnea	Atherosclerosis, hyperuricemia	Diabetes mellitus, hypertension, hyperlipidemia, hyperuricemia, sleep apnea	Atherosclerosis Pulmonary nodule	Atherosclerosis, impaired glucose tolerance, leukoencephalopathy
Clinical symptoms	Amnesia >1 year	Amnesia >14 years	Amnesia >1 year	Amnesia >1 year	Amnesia >10 years
	Neurobehavioral abnormalities for 1 year	Aphasia	Forgetfulness	Behavioral abnormalities >3 years	Forgetfulness
	Slow reaction	–	–	–	–
	Reduced ability in decision-making and comprehension		Loss of ability in decision-making	Reduced ability in verbal communication	
Staging of AD a	5C	6D	5C	6C	4C
Biomarkers of AD					
Aβ42	311.4	130.6	553.44	439.0	135.51
Aβ40	4,417.5	3,023.1	13,692.4	11,489.5	3,123.78
Aβ42/Aβ40	0.0705	0.0432	0.040	0.038	0.0434
p-Tau 217	13.1	103.9	–	–	70.5
P-Tau 181	–	–	48.37	52.3	/
NfL	3,904.3	1,342.4	–	–	1,124.4

Abbreviations: Aβ, amyloid-beta; AD, Alzheimer's disease; NfL, neurofilament light; p-Tau, phosphorylated tau.

a Disease staging is based on the classification proposed by the National Institute of Aging–Alzheimer's Association (NIA-AA). 5

### Biomarker Evaluation Using PET/CT Scan

**Fig. 6:**
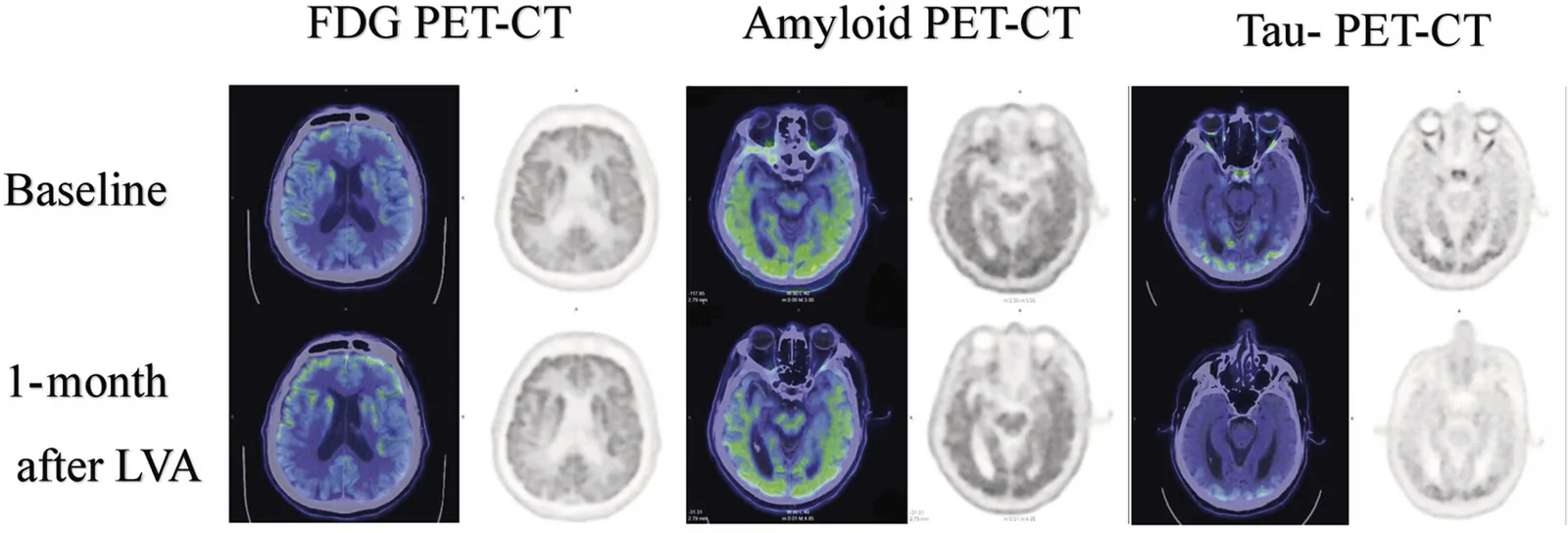
Trends of SUVr values across 19 brain regions on FDG-PET scans at baseline and postoperatively. FDG, fluorodeoxyglucose; PET, positron emission tomography; SUVr, standardized uptake value ratio.

Postoperative PET/CT imaging revealed varying degrees of enhancement in glucose metabolism and reductions in Aβ and tau protein deposits across 10 brain regions ( Figs. 6 7 8 ). Notably, the hippocampus demonstrated significant clinical responsiveness in various patients ( Fig. 9 ). Among these cases, patient 3 exhibited a 6.9% increase in FDG uptake, whereas reductions in amyloid and tau deposition were noted in patient 5 (12.3%) and patient 4 (4.69%), respectively.

**Fig. 7:**
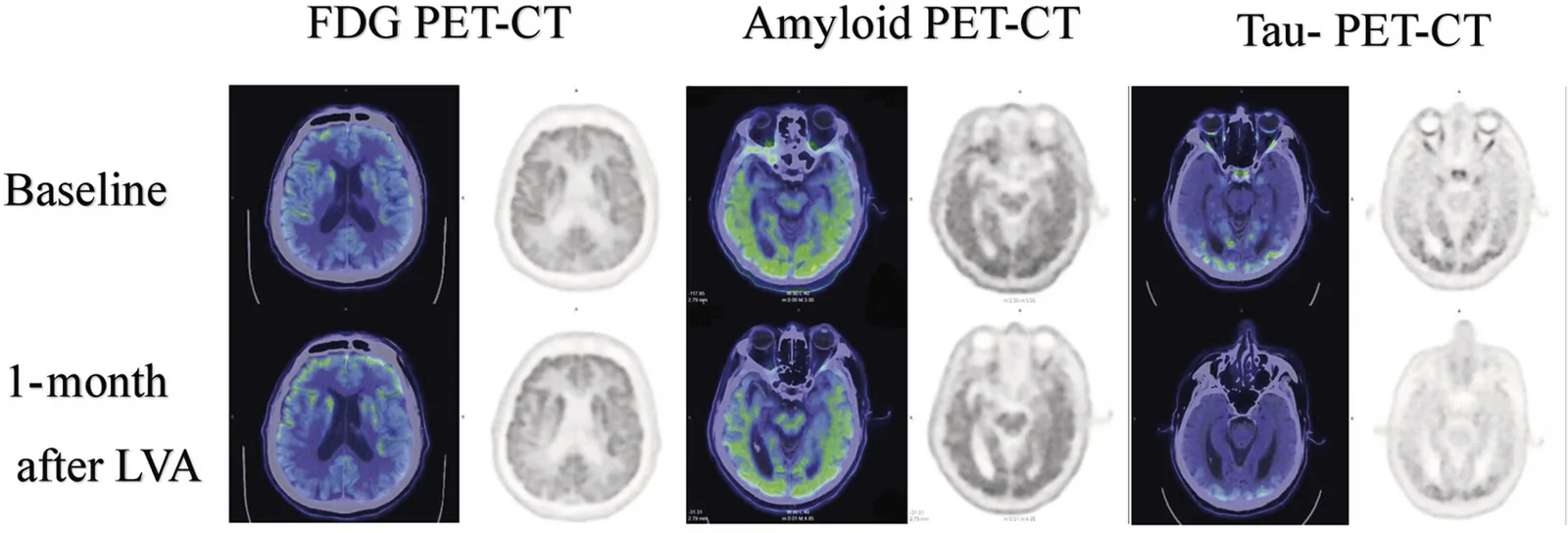
* Trends of SUVr values across 19 brain regions on amyloid-PET scans at baseline and postoperatively. AV45, florbetapir; SUVr, standardized uptake value ratio.

**Fig. 8:**
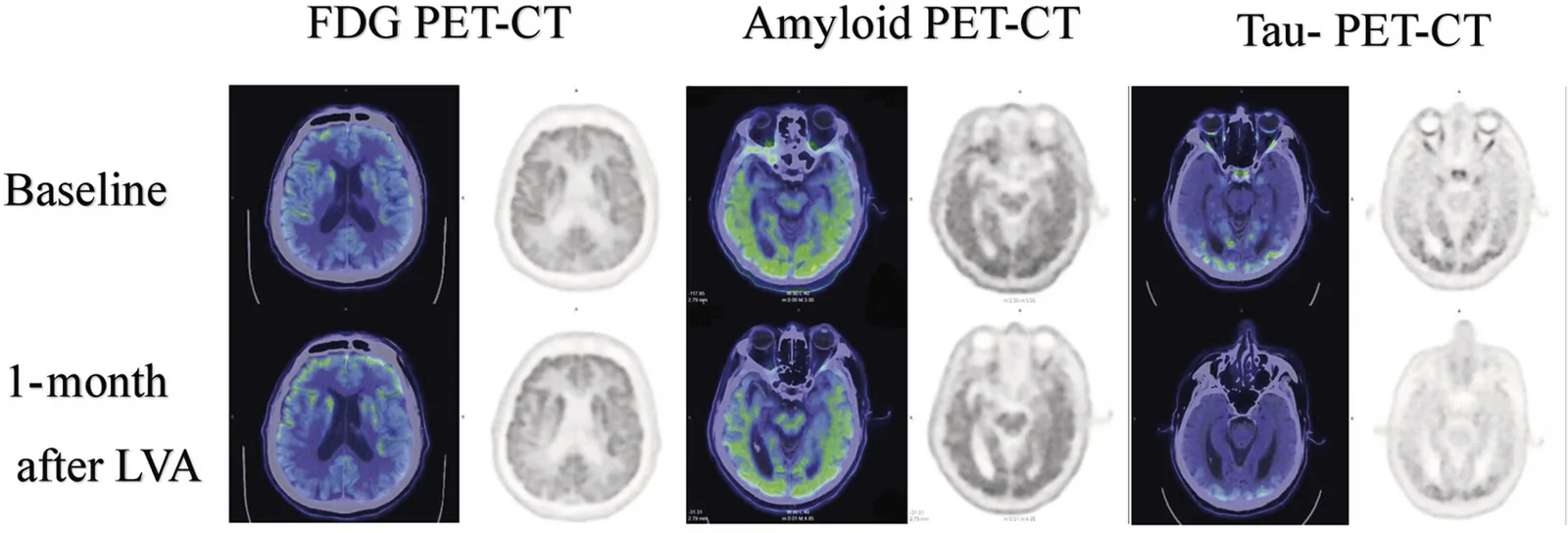
Trends of SUVr values across 19 brain regions on tau-PET scans at baseline and postoperatively. AV1451, flortaucipir; SUVr, standardized uptake value ratio.

**Fig. 9:**
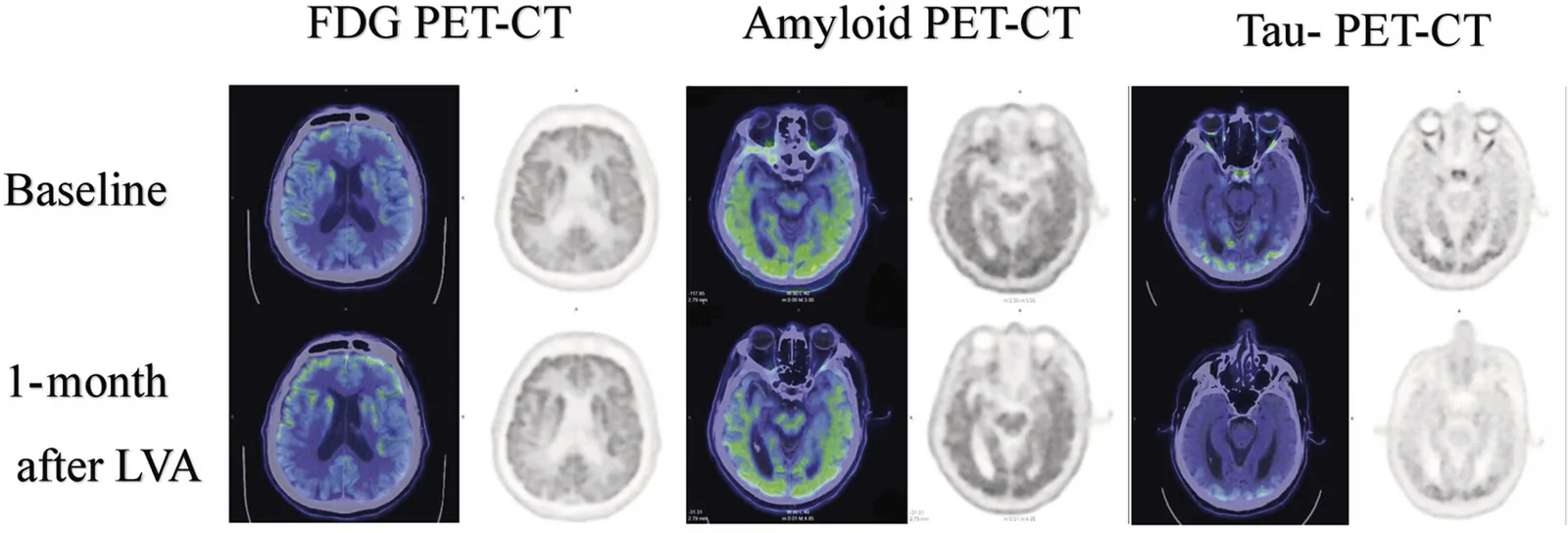
The PET-CT scans of subject 2 in three different configurations, FDG PET/CT, amyloid PET/CT, and tau-PET/CT, to evaluate glucose metabolism, the accumulation of amyloid and tau proteins, respectively. FDG, fluorodeoxyglucose; LVA, lymphovenous anastomosis; PET/CT, positron emission tomography/computed tomography.

### Clinical Evaluation Using Cognitive and Neuropsychological Tools

Cognitive and neuropsychological changes after DN-LVA are shown in Table 2 . Both MMSE and MoCA scores were improved by 1.8 and 1.4, respectively. NPI scores decreased by 5.8 across five subjects. The CDR remained consistent both before and after the procedure. Interviews with caregivers revealed subjective improvements in cognition and emotional state, and reduced caregiver distress and anxiety, which was reflected in NPI scores.

**Table 2:** Clinical outcomes of cognitive assessment conducted at baseline and 1 month postprocedure

	Subject 1	Subject 2	Subject 3	Subject 4	Subject 5
MMSE - Baseline - Postop	13 12	0 1	14 18	9 12	20 22
MoCA - Baseline - Postop	8 10	0 0	11 13	4 5	14 16
NPI - Baseline - Postop	12 10	44 22	1 3	13 –	1 0
CDR - Baseline - Postop	2 2	3 3	1 1	1 –	1 1

Abbreviations: CDR, Clinical Dementia Rating; MMSE, Mini-Mental State Examination; MoCA, Montreal Cognitive Assessment; NPI, Neuropsychiatric Inventory.

## Discussion

LVA has been extensively utilized in lymphedema management, demonstrating efficacy in resolving lymphatic obstruction. 14 This technique aims to create an alternative pathway by bypassing the blocked LV. Recent findings regarding the glymphatic clearance system 18 suggest that dysfunction in lymphatic clearance, facilitated by the water channel aquaporin-4 (AQP4), leads to the abnormal accumulation of Aβ and tau proteins. 19 20 21 AQP4-knockout Aβ precursor protein/presenilin 1 (APP/PS1) mice exhibited a 50% increase in Aβ accumulation. 21 These findings suggest that lymphatic dysfunction may play a role in the progression of AD and that restoration of lymphatic drainage could enhance cerebral waste clearance and potentially improve clinical outcomes. 10 12 13 The observed enhancement of cerebral lymphatic drainage following DN-LVA may be attributable to the creation of novel bypass pathways that partially circumvent conventional lymph node chains. In addition, pathological dilation of LVs beyond approximately 0.7 mm has been associated with valvular incompetence and reflux, resulting in impaired lymphatic circulation; LVA may mitigate this dysfunction by redirecting lymphatic flow into the venous system. In the present study, patients demonstrated clinical improvement in cognitive function, supporting a potential therapeutic role for cervical LVA in modulating cerebral lymphatic drainage.

Successful lymphaticovenous anastomosis is dependent on several key factors, including appropriate vessel caliber matching, meticulous intima-to-intima coaptation, the presence of functional venous valves, strategic planning of multiple LVAs, and optimization of the Venturi effect. 22 23 24 The EJV was selected as the recipient vessel because of its relatively superficial anatomical course compared with the IJV, which facilitates the creation of multiple end-to-end anastomoses and allows more reliable intraoperative assessment of anastomotic patency. During the procedure, we observed that intraluminal pressure within the IJV was relatively low, rendering direct anastomosis to LVs technically challenging for effective lymphatic inflow and potentially increasing the risk of venous backflow. During the dissection of the platysma muscle and deep cervical LVs, cervical cutaneous nerves may be encountered. All procedures were performed under an operating microscope, with meticulous microsurgical dissection to minimize manipulation and prevent injury to adjacent neural structures.

A key technical distinction of the DN-LVA technique compared with its predecessor is the use of a dorsalis pedis vein graft tunneled within the sternocleidomastoid muscle. This configuration permits the creation of multiple LVAs between distal LVs at the deeper aspect of the tunnel and the EJV superficially. The presence of intraluminal valves within the autologous vein graft, together with contraction of the sternocleidomastoid muscle during physiological head movement, is intended to simulate a muscular pump mechanism analogous to that of the lower limb. 25 This dynamic construct underpins the concept of DN-LVA and is designed to augment lymphatic flow while maintaining graft patency. In addition, the selection of the dorsalis pedis vein, which commonly exhibits multiple branches, facilitates multiple end-to-end anastomoses with distal LVs, thereby enhancing lymphatic drainage capacity. Furthermore, the Venturi effect—characterized as increased flow velocity and reduced intraluminal pressure within a narrowed conduit—is amplified by the use of a smaller-caliber vein graft relative to the EJV. Collectively, these refinements aim to promote unidirectional lymphatic flow, improve drainage efficiency, and reduce the risk of venous reflux. The term “dynamic,” as introduced in this study, is intended as a conceptual descriptor of the proposed physiological mechanisms. Future studies incorporating three-dimensional microbubble-enhanced super-resolution ultrasound imaging are planned to further characterize these dynamic features and to establish a more objective, operational, and reproducible framework for DN-LVA assessment.

The surgical outcomes of DN-LVA techniques were evaluated through cognitive evaluation tests and PET/CT imaging. CSF specimen analyses were conducted at baseline to confirm the diagnosis of AD; however, they were excluded from the clinical evaluation of surgical outcomes due to their invasive nature during collection, limited specificity in differentiating them from other dementias, and difficulties in predicting disease progression. The current understanding in the management of AD is that pharmacotherapy (e.g., cholinesterase inhibitors) and cognitive rehabilitation alone do not significantly modify the deposition of cerebral amyloid-β or tau proteins. Our results from the clinical cognitive assessment demonstrated a modest improvement in cognitive functioning. These assessment instruments offer a thorough appraisal of the patient's clinical condition, encompassing cognition, behavior, and activities of daily living. As illustrated in Fig. 7 , qualitative comparison of PET/CT images obtained at baseline and 1 month postoperatively was limited by patient motion and variability in head positioning during the imaging process. Consequently, quantitative analysis utilizing standardized uptake values derived from predefined regions of interest was conducted, enabling reproducible measurement of metabolic and protein deposition changes while minimizing observer-dependent variability. 26 To reduce potential confounding, pharmacologic regimens were maintained unchanged throughout the study period. The PET/CT findings indicate a greater reduction in amyloid plaques than in tau levels, predominantly within the hippocampal region. Although the underlying mechanism remains uncertain, this finding may relate to the differences in the localization and elimination patterns of both proteins. The propagation of amyloidosis and tauopathies follows a prion-like model, 27 in which extracellular amyloid deposition typically precedes and facilitates intracellular tau aggregation. 28 The present findings suggest that improvement in glymphatic clearance of solutes associated with LVA, without direct modulation of AQP4 function, may preferentially influence extracellular amyloid clearance while exerting a more limited effect on intracellular tau pathology. Nevertheless, the precise mechanism by which DN-LVA may modulate AD pathophysiology remains to be elucidated. These patients will be continuously monitored over prolonged follow-up periods of 12 months, 2 years, and 3 years in the future to evaluate the true effectiveness of LVA in managing AD patients.

This study identifies several limitations. The findings in this paper suggest potential for surgical interventions in AD; however, they remain inconclusive and require further follow-up and a larger sample size to determine any correlation between lymphatic reconstruction and symptomatic improvement in AD. Secondly, the procedure was conducted by the same surgeon at a single center without a control group, which limits the generalizability of the findings. Additional comparative studies are required to assess the effectiveness of this method in relation to other surgical techniques. The LVs selected for anastomosis in this study were located cranially to the identified deep cervical lymph nodes. It is still unclear if these lymph vessels represent the most effective option for LV augmentation in the treatment of atopic dermatitis. Further research is necessary to clarify the mechanisms underlying intracranial lymphatic drainage and to identify the optimal LVs for enhancing outcomes in LV anastomosis in AD.

### Conclusion

The DN-LVA presented in this paper holds some promise for potential utilization as an adjunctive management for AD. The selection of small-caliber vein grafts has allowed us to mitigate the risk of venous reflux while optimizing the patency of anastomosed vessels. Furthermore, this study represents an initial attempt to validate our modified surgical technique for a complex and poorly understood condition. Future research should include larger patient populations, prolonged observation durations, and randomized assignments to evaluate the long-term effectiveness of cervical LVA in the treatment of AD.
